# Nail Gun Injuries to the Hand and Wrist: A Systematic Literature Review

**DOI:** 10.7759/cureus.79216

**Published:** 2025-02-18

**Authors:** Prakash Chintapalli, Eimear Phoenix, Shane Cullen, Roisin T Dolan

**Affiliations:** 1 Plastic and Reconstructive Surgery, St. Vincent’s University Hospital, Dublin, IRL; 2 Department of Plastic and Reconstructive Surgery, Royal College of Surgeons in Ireland, Dublin, IRL

**Keywords:** hand functions, hand injury, hand trauma management, nail gun injury, rehabilitation

## Abstract

Nail gun injuries to the hand are common among those in the construction industry and are frequent presentations to the emergency department. A systematic review was conducted to analyze the long-term morbidity and functional outcomes following nail gun injuries to the hand. A literature search of four scientific databases was performed to identify articles describing nail gun injuries to the hand, up to and inclusive of October 2023. In total, 16 studies were eligible for inclusion, capturing 185 nail gun injuries to the hand. These included one major vascular injury, 23 nerve injuries, 46 tendon injuries, and 36 bone injuries. Morbidity on follow-up included two cases of motor dysfunction, 23 cases of chronic pain or paresthesia, and 11 cases of reduced range of motion. Nail gun injuries to the hand require thorough evaluation techniques, an understanding of surgical versus non-surgical management, and close postoperative monitoring and rehabilitation.

## Introduction and background

First introduced in 1959, nail guns are powerful tools used to efficiently drive nails into wood, metal, and concrete. Powered by compressed air, an explosive charge, or electricity, this tool generates forces capable of propelling projectiles as large as 10 cm in length to velocities up to 426 m per second. While used primarily by construction workers in framing, they are increasingly used by the general public [1]. With this rise in use, the number of injuries involving nail guns has risen significantly [2,3]. The most common site of injury is the hand [4]. The radial aspect of the non-dominant hand is most susceptible to injury, as it is often used to position the object to be nailed [5]. Injuries commonly occur due to careless handling of equipment and accidental discharge, although some involve overpenetration of structures by the projectile and the ricochet or shattering of the projectile [6].

Nail gun projectiles may penetrate structures such as nerves, tendons, major vessels, or bone. As a result, there is significant potential for long-standing morbidity, including pain, sensorimotor deficits, and stiffness. However, few studies report on the outcomes of nail gun injuries to the hand and wrist, and no systematic reviews have been conducted on this topic to our knowledge. This systematic review aimed to analyze the current literature regarding the outcomes of nail gun injuries. The review evaluated the frequency of structural injury and the subsequent effects present on follow-up.

This article was previously presented at the Sylvester O’Halloran Perioperative Symposium from the 29th of February to the 2nd of March 2024 and at the Federation of European Societies for the Surgery of the Hand on 26-29 June 2024 meetings as a poster.

## Review

Methodology

A systematic review of the literature was conducted in accordance with the Preferred Reporting Items for Systematic Reviews and Meta-Analyses (PRISMA) guidelines [7]. The review protocol was registered with the PROSPERO systematic review database (CRD42023468404).

Eligibility Criteria

To be eligible for this systematic review, a study must have reported injury to the hand or wrist from a nail gun in a child or adult, with no limitations placed on language. Characteristics for inclusion were articles published in peer-reviewed journals to ensure reliable methodology. The study period was from 30 September 2023 to 30 October 2023.

Information Sources and Search

Medline, PubMed, Scopus, and the Cochrane Library were searched for identifying relevant articles. All articles meeting the inclusion criteria were reviewed. Study publication dates ranged from 1965 to 2015. For PubMed, the search was conducted using the advanced search option. The keywords “nail gun” or “nail-gun” or “nailer” or “stud gun” AND “hand” (title/abstract) were used. The search was conducted exclusively by the principal author and decisions were reviewed by the second author.

Inclusion and Exclusion Criteria

**Figure 1:**
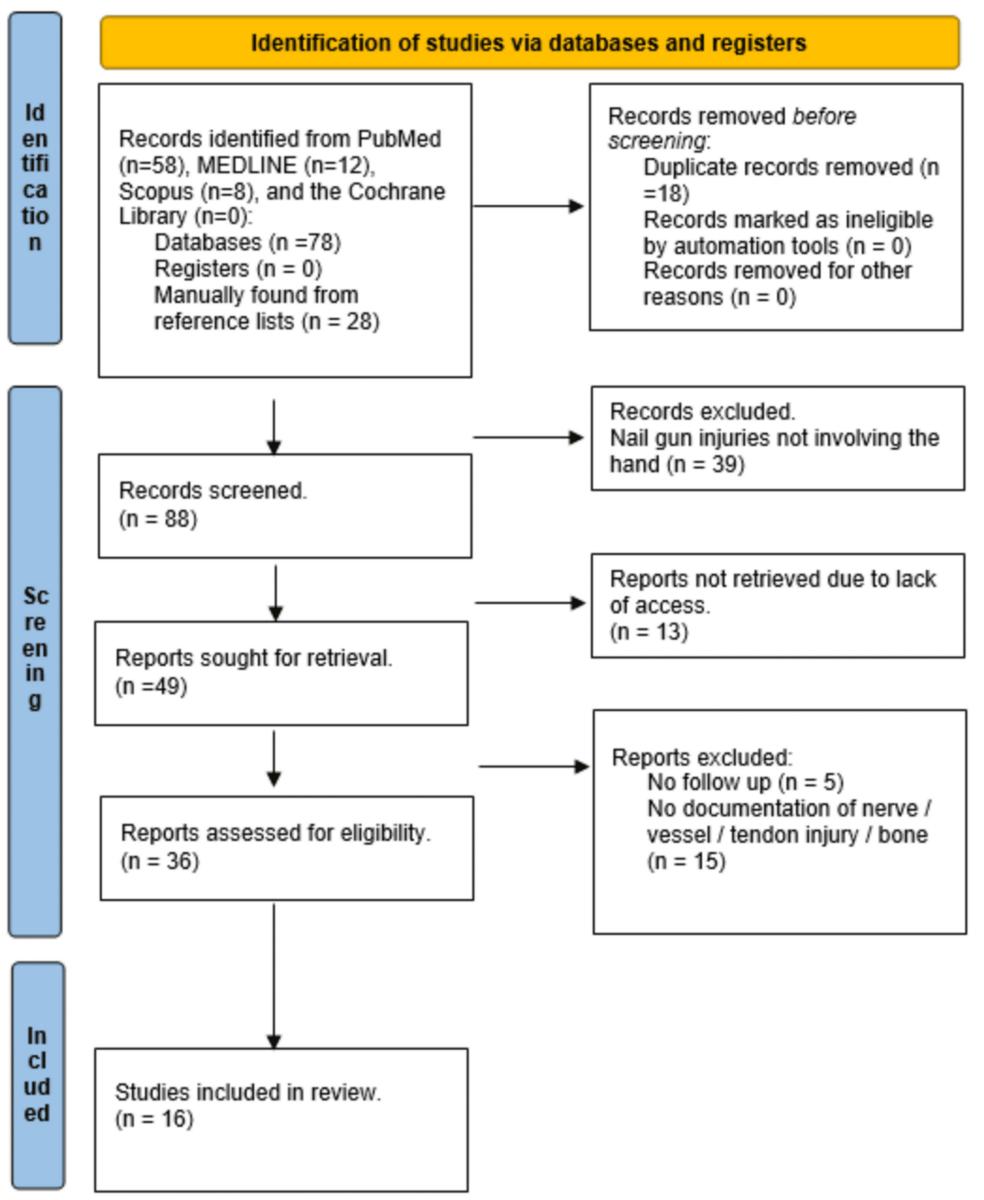
Preferred Reporting Items for Systematic Reviews and Meta-Analyses (PRISMA) flow diagram of study selection.

We included articles that (1) had full-text availability; (2) involved nail gun injury to the hand or wrist in children or adults (3) where injury to tendons, bone, nerve, or vessel was recorded; and (4) had at least one-week follow-up of clinical outcomes for stiffness and change in sensation or motor function. Studies that did not meet all the inclusion criteria were excluded. Figure 1 demonstrates the PRISMA flow diagram detailing the search process.

Study Selection and Data Extraction

Two independent reviewers (EP and SC) reviewed all titles and abstracts, and those eligible for inclusion were entered into a database. After this, all duplicates were eliminated. Articles deemed suitable for inclusion were retrieved as full-length papers and assessed by the same two reviewers. Disagreements were resolved by consensus, or, if necessary, with arbitration by the senior author (RD).

A data extraction table was formulated to include the following information from each article: authors; journal; year of publication; title; study type; geographical location; number of patients; a record of injury to nerve, tendon, bone, or vessel; intervention; outcome of persisting change in the sensation, pain, stiffness, reduced range of motion, and reduced motor function on follow-up.

Appraisal and Synthesis of Results

The data extracted from each study were tabulated by authors; year of publication; number of nail gun injuries to the hand included; presence of injury to the nerve, vessel, tendon, or bone; and follow-up outcome of persisting deficit in sensation, persisting pain, motor deficit, and range of motion deficit. We examined the frequency of these injuries and their outcomes in each study.

Risk of Bias in Individual Studies and Across All Studies

The Joanna Briggs Institute (JBI) critical appraisal checklists for case series and the checklist for case reports [8] were employed to assess the risk of bias and the results were tabulated (Tables 1, 2) [9,10]. Overall, only one case series by Stoebner et al. [9] and two case reports scored satisfactorily on the entirety of the checklist’s questions. All included series had clear criteria for inclusion, correctly identified the initial injury in a standard fashion, and used valid methods for the identification of the nail gun injury (Q1-3). Four of the 10 included case series did not have consecutive inclusion of participants (Q4), and six of 10 did not have complete inclusion of participants (Q5). Generally, there was clear reporting of clinical information (Q7); however, follow-up outcomes were only clearly reported in four series (Q8). Four studies reported their clinical site’s demographic information (Q9), and statistical analysis was generally not applicable in most of the series included due to the low number (Q10). This critical appraisal highlights poor reporting of outcomes in the literature concerning nail gun injury outcomes [1-34].

**Table 1:** Joanna Briggs Institute (JBI) critical appraisal checklist for case series. NA: not applicable

Authors	Paper title	Q1	Q2	Q3	Q4	Q5	Q6	Q7	Q8	Q9	Q10	Total yes
Horne and Corley, 2008 [2]	Review of 88 nail gun injuries to the extremities	Yes	Yes	Yes	Yes	No	Yes	No	No	Yes	No	6
Pierpont et al., 2008 [3]	Nail-gun injuries to the hand	Yes	Yes	Yes	No	No	Yes	Yes	Yes	No	NA	6
Kenny et al., 1993 [6]	Nail gun injuries	Yes	Yes	Yes	No	Unclear	Yes	Yes	Yes	No	NA	6
Stoebner et al., 2011 [9]	Upper and lower extremity nerve injuries in pediatric missile wounds: a selective approach to management	Yes	Yes	Yes	Yes	Yes	Yes	Yes	Yes	Yes	Yes	10
Ling et al., 2013 [11]	Eighty-seven cases of a nail gun injury to the extremity	Yes	No	Yes	Yes	No	No	No	Unclear	Yes	Yes	5
Hussey et al., 2008 [12]	Nail gun injuries to the hand	Yes	Yes	Yes	Yes	No	Yes	Unclear	Unclear	Yes	Yes	7
Goldin and Economou, 1965 [15]	Stud gun injuries	Yes	Yes	Yes	No	Unclear	Yes	Yes	No	No	NA	5
Ridha et al., 2002 [16]	Nail-gun limb injuries	Yes	Yes	Yes	Unclear	Unclear	Yes	Yes	Unclear	No	NA	5
Paton et al., 1986 [17]	Injuries from nail gun cartridges: a dangerous new game	Yes	Yes	Yes	No	Unclear	Yes	Yes	Yes	No	NA	6
Van Demark Jr et al., 1993 [31]	Nailgun injuries of the hand	Yes	Yes	Yes	No	No	Yes	Yes	Unclear	No	NA	5

**Table 2:** Joanna Briggs Institute (JBI) critical appraisal checklist for case reports.

Authors	Paper title	Q1	Q2	Q3	Q4	Q5	Q6	Q7	Q8	Total yes
Li et al., 2012 [10]	The effective analysis of microsurgical repair of radial nerve deep branch injury	Yes	Yes	Yes	Yes	Yes	Yes	Yes	Yes	8
Bitzos et al., 2009 [13]	Management of penetrating wrist injuries in the emergency department	Yes	Yes	Yes	Yes	Yes	Yes	Yes	Yes	8
Freeman et al., 1993 [14]	Nail gun injury: an update	Yes	Yes	Yes	Yes	Yes	No	Yes	Yes	7
Tong et al., 2011 [29]	Use of a hand drill to remove particulate contamination within a traumatic bone tunnel	Yes	Yes	No	Yes	Yes	No	No	Yes	5
Boya and Uzun, 2015 [33]	Hand injury with a nail gun: a case report and literature review	Yes	Yes	Yes	Yes	Yes	No	Yes	Yes	7
Wilson 1962 [34]	Industrial bullet wound	Yes	No	Yes	Yes	Yes	No	No	Yes	5

Results

Study Selection

The electronic database search yielded 78 studies. Reference lists of these studies were screened for other relevant articles, yielding 28 further studies. After duplicates were removed and inclusion criteria were applied, 16 studies were eligible for review (Figure 1).

Study Characteristics

Table 3 demonstrates the characteristics of the included studies. A total of 185 patients were included across the 16 studies. Of these, 10 were case series and six were case studies. Overall, 23 nerve injuries, one vascular injury, 46 tendon injuries, and 39 bony injuries were reported. Surgical management was employed in 132 cases, and the use of intravenous (IV) antibiotics and anti-tetanus was standard. Follow-up periods ranged from 17 days to 39 months, showing huge variance and the lack of an established protocol for follow-up in nail gun injuries.

**Table 3:** Summary of studies including the rates of structure injury and functional status at follow-up. ^1^: Of 88 patients, 34 had a hand or finger injury, eight of 24 had ongoing pain at follow-up, and two of 24 had ongoing paraesthesia. The bony injury rate of 25% was extrapolated to obtain the number affected. The median nerve was compressed by edema due to the nail, but not directly penetrated. NR: not recorded; ROM: range of motion

Authors	Paper title	N	Injury				Deficits on follow-up		
			Nerve	Vascular	Tendon	Bone	Sensory	Motor	ROM
Horne and Corley 2008 [2]	Review of 88 nail gun injuries to the extremities	34^1^	2	0	NR	8	2	0	0
Pierpont et al., 2008 [3]	Nail-gun injuries to the hand	2	0	0	0	0	0	0	0
Kenny et al., 1993 [6]	Nail gun injuries	3	0	0	0	0	0	0	0
Stoebner et al., 2011 [9]	Upper and lower extremity nerve injuries in pediatric missile wounds: a selective approach to management	1	1	0	0	0	1	0	0
Li et al., 2012 [10]	The effective analysis of microsurgical repair of radial nerve deep branch injury	3	3	NR	0	NR	0	1	0
Ling et al., 2013 [11]	Eighty-seven cases of a nail gun injury to the extremity	64	14	0	32	21	3	0	1
Hussey et al., 2008 [12]	Nail gun injuries to the hand	55	2	0	10	2	8	0	6
Bitzos et al., 2009 [13]	Management of penetrating wrist injuries in the emergency department	1	0	1	0	0	0	0	0
Freeman et al., 1993 [14]	Nail gun injury: an update	1	0	0	1	1	0	0	0
Goldin and Economou, 1965 [15]	Stud gun injuries	5	1	0	1	1	1	1	3
Ridha et al., 2002 [16]	Nail-gun limb injuries	3	0	0	1	1	0	0	0
Paton et al., 1986 [17]	Injuries from nail gun cartridges: a dangerous new game	2	0	0	1	0	0	0	0
Tong et al., 2011 [29]	Use of a hand drill to remove particulate contamination within a traumatic bone tunnel	2	0	0	0	2	0	0	0
Van Demark Jr et al., 1993 [31]	Nail gun injuries of the hand	7	0	0	0	2	0	0	1
Boya and Uzun, 2015 [33]	Hand injury with a nail gun: a case report and literature review	1	0	0	0	0	0	0	0
Wilson, 1962 [34]	Industrial bullet wound	1	0	0	0	1	0	0	0

Nerve Injury

Of the 185 cases with reported follow-up outcomes, 23 involved potential nerve injury by a nail gun (Table 3). The classification of what constituted nerve injury was variable between studies. Standard measures such as the Ten Test and MRC Muscle Power Scale were employed only by Stoebner et al. [9] and Li et al. [10]. The remaining series classified nerve injury based on either symptoms of paresthesia or only on objective intraoperative findings of nerve injury. With this variation, overall nerve injury rates were reported at 16%, 4%, and 6% by the three largest case series by Ling et al. [11], Hussey et al. [12], and Horne and Corley [2], respectively. It is difficult to ascertain a true nerve injury rate in nail gun trauma with the data available, as paresthesia may be caused by swelling at the injured sites without nerve damage. Estimation based on operative findings and ongoing paresthesia at follow-up provides a nerve injury rate of 3.9%.

Vascular Injury

Major vascular injury was exceedingly rare, and of the included studies, only one case report by Bitzos et al. [13] found a significant vascular injury. This involved perforation of the radial artery, which was managed with segmental excision and anastomosis.

Tendon Injury

Tendon involvement from nail gun injury was reported in 46 cases, with 32 reported by Ling et al. [11]. Tendon injury is reported inconsistently, and overall tendon injury rate by nail gun is likely between 18% and 25% of all nail gun injuries. Ling et al. [11] and Horne and Corley [2] did not categorize tendon injuries separately, instead reporting a number including tendon, joint capsule, and neurovascular injury together [2,11], deriving a tendon injury rate of close to 25%. A clearer picture emerged from the study by Hussey et al. [12]. Of the 55 nail gun injuries to the hand, 10 had tendon injuries, giving a rate of 18%. A review of case reports [14-17] showed that extensor tendon injuries were more frequently reported than flexor tendon injuries. Three case reports involved extensor tendon injury, and only one flexor tendon injury was reported.

Bony Injury

We found 37 documented cases of bony injury by a nail gun to the hand. The classification of bony injury is heterogeneous, with Ling et al. [11] reporting bone involvement in 21 cases but noting no fractures. Horne and Corley [2] reported that 52.3% of nail gun injuries recorded involved bony penetration but did not specify which cases were related only to hand and wrist nail gun injuries. No cases required bone fixation techniques. Hussey et al. [12] reported two cases of bone penetration, with neither requiring fixation. No case studies or smaller series reported bony injury by nail gun requiring fracture fixation. Overall, the bony injury rate was best estimated using the study by Hussey et al. [12] at 3.6%. While much higher rates are suggested by other case series, no detail regarding the distribution of bone injury specifically in the hand has been described.

Follow-Up and Outcomes

Follow-up of nail gun injuries was varied, with the literature showing many patients lost to follow-up due to absenteeism from booked appointments [11,12]. Just under two-thirds of patients with nail gun injuries were followed up beyond their initial presentation. Their duration of follow-up is demonstrated in Table 4 and ranges from a few days up to 39 months, with the median duration being three weeks. In five studies, the duration was not specified although outcomes at follow-up were described.

**Table 4:** Management, follow-up duration, and nail characteristics. ^1^: In the study by Horne and Corley, percentages rather than numbers were used to report outcome measures. As this paper included both upper and lower limb injuries, we cannot extrapolate the exact figures pertinent to hand and wrist nail gun injuries. NR: not reported

Authors	N	Management				Follow-up		Nail characteristics
		Operative	Non-operative	IV abx	Tetanus	N	Follow-up duration (weeks)	
Horne and Corley, 2008^1^ [2]	34	NR (33% of cases)	NR (67% in the emergency room)	NR (84%)	NR	NR. 27.3% of cases (42% of those who underwent operative management)	1.75	NR
Pierpont et al., 2008 [3]	2	2	0	NR	NR	2	Duration NR	Barbs
Kenny et al., 1993 [6]	3	3	0	NR	NR	3	Duration NR	Barbs
Stoebner et al., 2011 [9]	1	1	0	1	1	1	52	NR
Li., et al 2012 [10]	3	3	0	3	3	3	48–468	NR
Ling et al., 2013 [11]	64	50	14	50	64	30	0.1–2.25	NR
Hussey et al., 2008 [12]	55	55	0	55	55	52	0.1–26	NR
Bitzos et al., 2009 [13]	1	1	0	1	1	1	Duration NR	No barbs
Freeman et al., 1993 [14]	1	1	0	1	1	1	6	Barbs
Goldin and Economou 1965 [15]	5	5	0	5	NR	5	12	NR
Ridha et al., 2002 [16]	3	3	0	NR	NR	3	12	Threaded
Paton et al., 1986 [17]	2	2	0	2	NR	2	Duration NR	No barbs (cartridge injury)
Tong et al., 2011 [29]	2	2	0	NR	NR	2	8–12	No barbs
Van DemarkJr et al., 1993 [31]	7	2	0	2	NR	2	16–64	NR
Boya and Uzun 2015 [33]	1	1	0	1	1	1	48	No barbs
Wilson 1962 [34]	1	1	0	NR	NR	1	Duration NR	No barbs, plastic sleeve

Outcomes have been reported sparsely in the literature, and rarely with the use of standardized measures of nerve or range of motion deficits such as the MRC power scale and Ten Test. Five studies of documented nail gun injury [18-22] did not report follow-up outcomes (Table 5) and could not be included for analysis in this review despite describing nail gun injuries specifically. The use of hand therapy was reported in only four studies [2,3,9,12]. These limitations have the potential to result in the underreporting of persistent deficits related to nail gun injuries.

**Table 5:** Summart of studies including the rates of structure injury without follow-up.

Authors	Paper title	N	Injury			
			Nerve	Vascular	Tendon	Bone
Lanteri et al., 2012 [18]	Barbed screw through the hand	1	1	0	1	0
Knott et al., 2004 [19]	Nail gun injuries presenting to the emergency department	227	2 (0.9%)	0	17 (7.6%)	48 (21%)
Armstrong and Davies, 1996 [20]	Nail gun injury: a barbed problem	1	0	0	0	0
Al Qattan and Stranc, 1993 [21]	Nail gun injuries of the finger: a safer method of removal	1	0	0	0	0
Lyons 1983 [22]	Industrial nail gun injuries	1	0	0	0	1

Sensory deficits persisting on follow-up were reported in 15 cases, motor deficits in two cases, and stiffness in 11 cases (Table 3). These remarkably low numbers may be related to inconsistent follow-up and low duration of follow-up in many cases, as described above. Pooled analysis of the largest three case series provides rates of 8% with sensory deficits on follow-up, 0.6% with motor deficits, and 4.5% with stiffness.

Discussion

Nail guns are a commonly used tool in the construction industry and have now become readily available to consumers. Most are powered by a pneumatic system in which compressed air moves a piston to drive the nail forward, while a less common mechanism involves the use of a small explosive charge to propel the nail [3]. Of the 37,000 nail gun-related injuries found annually in the United States, 40% were in consumers [1] who receive little, if any, training on the use of these powerful tools and are over three times more likely to injure themselves compared to experienced workers [23]. The mechanism of injury is varied, including penetrating injury by the projectile, cartridge shrapnel, and high-pressure injection injury by the compressed air used in pneumatic nail guns. Here, we discuss a systematic approach to the assessment and management of nail gun injuries in the hand. The first step lies in understanding the capabilities of the nail gun and the characteristics of its projectile.

Nail Gun Characteristics

Two kinds of nail guns are widely available, i.e., the pneumatic type and the explosive cartridge-powered type. Pneumatic nail guns propel nails at a lower velocity (293 feet per second) [2,22] compared to the explosive charge-powered type (1,400 feet per second) [22]. Driven nails range from 5 × 2.5 cm to 8.9 × 0.41 cm in dimension but typically have the same head size. The kinetic injury of the penetrating nail is transferred to local tissue, with higher energy injuries causing cavitations in the tissue [3,4,24] and the lower velocity pneumatic guns typically causing injuries limited to the injury tract alone. The nails themselves may be bound by metal wires or resin which can remain in soft tissues or cause further injury on removal [23,25]. Other foreign bodies such as clothing fabric may be driven into wounds by nails on the head increasing the difficulty of decontamination [3].

Assessment

Meticulous clinical evaluation with the aid of radiological investigation guides the treating doctor toward a successful treatment plan. The nail should be left in place until the clinical and radiological evaluation is completed [4]. Local anesthesia should also be withheld until neurovascular examination is complete [4].

History taking should clearly establish the time of injury, angle of entry, and type of nail gun and nail used. If possible, other nails the patient may have brought should be examined to ascertain their characteristics. Any first aid administered should be noted, along with establishing a background medical history, hand dominance, smoking status, and occupation. Thorough interrogation should be done for symptoms of injury to the nerve and tendons and administering local anesthetics before examination should be avoided.

A systematic physical examination should be performed. Nail position, presence of barbs, deformity, abnormal hand posture, and proximity to hand structures, such as nerves, vessels, and tendons, should be inspected. Temperature, radial and ulnar pulses, capillary refill time, and sensory deficits in the distribution of the radial, median, and ulnar nerve should be noted. Next, the range of active and passive movement at relevant joints must be noted, along with motor deficits and possible tendon injury limiting movement. It is also important to examine the nail itself to estimate the size and the presence of threading, barbs, and resin.

Injuries should be classified according to the structures involved, including soft tissue only, nerves, vessels, tendons, joints, and bones [12]. Thorough neurovascular and tendon evaluation is crucial in differentiating patients who require simple wound care under local anesthetic versus an exploration under general anesthetic with tendon, nerve, or microvascular repair [4].

Radiographs, with a minimum of two views, are invaluable in the management of nail gun injury to rule out fractures and the presence of other foreign bodies that may have been driven into the hand [4].

**Figure 2:**
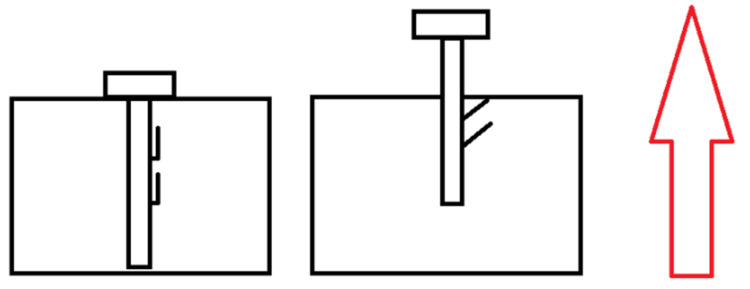
Barbs engaging on retrograde retrieval. Image credits: Satya Chintapalli.

The common presence of barbs [14,20] should be carefully noted, as these will require special consideration for nail extraction. Devastating soft tissue damage can be inflicted by pulling out a barbed nail in the wrong direction [2,14] (Figure 2). Barbs may trap salvageable nerves or vessels which can be inadvertently injured if the nail is removed without appropriate dissection. Any barbs embedded in bone can have a similar effect, creating comminuted fractures unnecessarily. As barbs are not always visible on X-rays, caution must be exercised during management despite radiological clearance [6,12].

Management

Management can be divided into operative and non-operative options depending on assessment. In any case, standard principles for the management of trauma should be maintained with nail gun injuries to the hand [26]. Prophylactic antibiotics are routinely used, and the need for tetanus immunization should be considered and updated if necessary [3].

Non-operative management with simple extraction, irrigation, and minimal debridement appears effective in cases with no evidence of structural injury, no limitation in range of motion, minimal contamination, and no intra-articular penetration or neurovascular compromise [2,4].

Most uncomplicated nails are removed in the emergency department (33% intraoperatively vs. 66% in the emergency department). Horne and Corley [2] found a low frequency of infection and rapid return to function for the majority of uncomplicated cases. They suggested that nail gun injuries to the extremities can be managed with simple extraction and minimal debridement in the emergency department when there is soft tissue injury alone.

If an exit is wound present, the nail should be cut flush with the entrance wound and removed in an antegrade fashion [27], but keep the risk of retained foreign body in mind [28]. If there is no exit wound, careful radiographic evaluation should dictate the direction of nail extraction, as the retrograde movement of the nail can deploy barbs, resulting in soft tissue injury [14]. This scenario may warrant delivering the nail in an anterograde fashion through a surgically created wound [4].

Stable fractures without substantial bone loss or contamination can be treated non-operatively with reduction and external fixation [26]. Operative management with surgical exploration is indicated in cases with high levels of contamination or devitalized tissue, neurovascular deficits, unstable fractures, intra-articular fractures, or tendon injuries [4].

Contamination may present in the form of retained foreign bodies, some of which remain difficult to dislodge with washout alone. Tong et al. described the use of hand drilling with copious irrigation in such cases [29]. Drains or cannulas threaded through the tract have also been described as innovative methods to allow targeted irrigation [30,31]. Other injuries to nerves, vessels, bones, and tendons can be treated in a targeted fashion after thorough irrigation and debridement or excision of structures within the zone of injury. Adequate dissection around the nail is crucial to ensure vital soft tissues do not remain trapped, suffering an iatrogenic injury during extraction. Definitive structural repair can be performed as a single-stage operation or in a staged fashion if abundant contamination is present. Similarly, wound closure is dictated by the extent of injury and degree of contamination.

**Figure 3:**
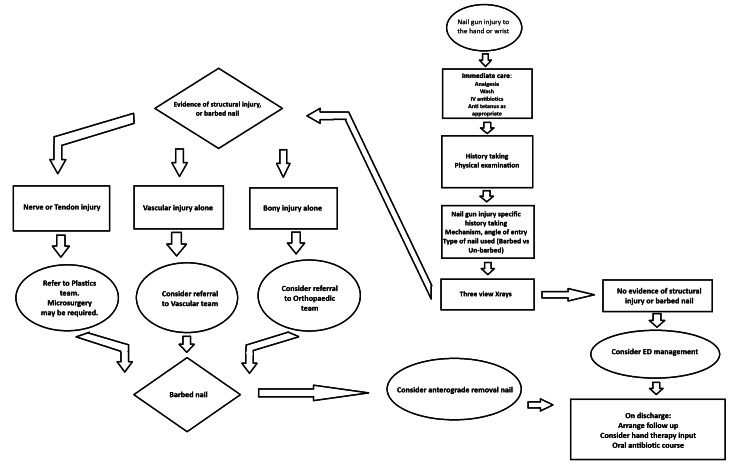
Suggested management algorithm for nail gun injuries to the hand and wrist. Image credits: Satya Chintapalli.

Regardless of the form of treatment, doctors should be conscious of preventing infection as the top priority. Antibiotics with anti-staphylococcal and anti-pseudomonal cover are recommended for five to seven days after extraction of the nail. Postoperatively, we suggest that all cases of tendon or bony injury requiring hand splinting should be followed up with hand therapy input. Figure 3 displays our suggested management algorithm.

Outcomes

As discussed above, outcomes are poorly reported in the literature. Overall, most nail gun injuries to the upper limb appear to have an uncomplicated course and are not associated with neurovascular or tendon injury. Table 3 presents 16 articles detailing cases of nerve, tendon, vascular, or bony injuries and the outcomes following the same on follow-up. Persistent issues on follow-up were most commonly due to sensory deficits, found in 8% of nail gun injury cases, followed by stiffness in 4.5%. Motor deficits appear rare on follow-up, with a rate of only 0.6%.

Follow-up duration was inconsistent and ranged from days to years in the literature. Those returning for follow-up were more likely to have a more complex injury or ongoing symptoms, and many patients were lost to follow-up due to absenteeism, resulting in an overall follow-up rate of 65%. Hand therapy input is not routinely sought but may significantly improve outcomes [32].

Limitations

Limitations of this review include a high risk of selection and reporting bias due to the exclusive use of case reports or series, and the paucity of other study types available. A significant proportion (26.5%) of studies intended for inclusion were unavailable for retrieval. Reporting of follow-up and injury classification was heterogeneous, and the overall captured number of cases was low in proportion to the frequency of this mechanism of injury.

## Conclusions

Nail gun injuries are an increasingly common problem due to the rise in the use of these powerful tools. These injuries have the potential to result in persistent sensory deficits and stiffness in many patients and must be managed with caution to avoid iatrogenic injury on nail extraction. We recommend a systematic approach to the assessment and management of nail gun injuries to the hand and wrist, involving careful history taking, physical examination, use of radiography, and appropriate surgical technique taking account of nail characteristics. The presence of barbs is a complicating factor that may lead to inadvertent iatrogenic injury and must be taken into account when planning a surgical approach. Further research into the follow-up and outcomes of these injuries is required, as is an increased awareness of the need for appropriate follow-up and hand therapy input.

## References

[1] (2007). Nail-gun injuries treated in emergency departments--United States, 2001-2005. MMWR Morb Mortal Wkly Rep.

[2] Horne BR, Corley FG (2008). Review of 88 nail gun injuries to the extremities. Injury.

[3] Pierpont YN, Pappas-Politis E, Naidu DK, Salas RE, Johnson EL, Payne WG (2008). Nail-gun injuries to the hand. Eplasty.

[4] Rhee PC, Fox TJ, Kakar S (2013). Nail gun injuries to the hand. J Hand Surg Am.

[5] Braun RM (1971). Nail-gun injury of the hand. J Bone Joint Surg Am.

[6] Kenny N, O'Donaghue D, Haines J (1993). Nail gun injuries. J Trauma.

[7] Liberati A, Altman DG, Tetzlaff J (2009). The PRISMA statement for reporting systematic reviews and meta-analyses of studies that evaluate health care interventions: explanation and elaboration. PLoS Med.

[8] Munn Z, Barker TH, Moola S (2020). Methodological quality of case series studies: an introduction to the JBI critical appraisal tool. JBI Evid Synth.

[9] Stoebner AA, Sachanandani NS, Borschel GH (2011). Upper and lower extremity nerve injuries in pediatric missile wounds: a selective approach to management. Pediatr Surg Int.

[10] Li J, Hu H, Wang H, Cheng X (2012). [The effective analysis of microsurgical repair of radial nerve deep branch injury]. Zhongguo Xiu Fu Chong Jian Wai Ke Za Zhi.

[11] Ling SN, Ong NC, North JB (2013). Eighty-seven cases of a nail gun injury to the extremity. Emerg Med Australas.

[12] Hussey K, Knox D, Lambah A, Curnier AP, Holmes JD, Davies M (2008). Nail gun injuries to the hand. J Trauma.

[13] Bitzos IE, Granick MS (2009). Management of penetrating wrist injuries in the emergency department. Eplasty.

[14] Freeman BJ, Ainscow DA (1994). Nail gun injury: an update. Injury.

[15] Goldin MD, Economou SG (1965). Stud gun injuries. J Trauma.

[16] Ridha H, Orakzai SH, Kearns SR, Roche-Nagle G, Keogh P, O'Flanagan SJ (2002). Nail-gun limb injuries. Ir Med J.

[17] Paton RW (1986). Injuries from nail gun cartridges: a dangerous new game. Br Med J (Clin Res Ed).

[18] Lanteri A, Celestin R, Fleegler E (2012). Barbed screw through the hand. Eplasty.

[19] Knott JC, Meyer AD, Kas P (2004). Nail gun-related injuries presenting to the emergency department. Emerg Med Australas.

[20] Armstrong AP, Davies DM (1996). Nail gun injury: a barbed problem. J Accid Emerg Med.

[21] al-Qattan MM, Stranc MF (1993). Nail gun injuries of the fingers: a safer method of nail removal. J Hand Surg Br.

[22] Lyons FR (1983). Industrial nail gun injuries. Med J Aust.

[23] Lipscomb HJ, Dement JM, Nolan J, Patterson D, Li L (2003). Nail gun injuries in residential carpentry: lessons from active injury surveillance. Inj Prev.

[24] Fackler ML, Bellamy RF, Malinowski JA (1988). The wound profile: illustration of the missile-tissue interaction. J Trauma.

[25] Kenny NW, Kay PR, Haines JF (1992). Nail gun injuries to the hand. J Hand Surg Br.

[26] Nathan R (1999). The management of penetrating trauma to the hand. Hand Clin.

[27] Absoud EM, Harrop SN (1984). Hand injuries at work. J Hand Surg Br.

[28] Kenny NW (1994). Safer method of removing the nail after a nail gun injury to the fingers. J Hand Surg Br.

[29] Tong PY, Cheah AE, Peng YP (2011). Use of a hand drill to remove particulate contamination within a traumatic bone tunnel. Hand Surg.

[30] Malahias M (2008). Nail gun injuries: a simple method to facilitate exploration. Ann R Coll Surg Engl.

[31] Van Demark RE Jr, Van Demark RE Sr (1993). Nailgun injuries of the hand. J Orthop Trauma.

[32] Stralka SW (2016). Hand therapy treatment. Hand Clin.

[33] Boya H, Uzun B (2015). Hand injury with a nail gun: a case report with literature review. Acta Orthop Traumatol Turc.

[34] Wilson PJ (1962). Industrial-bullet wound. Br Med J.

